# Supplementary material for: Agglomeration costs limit sustainable innovation in cities in developing economies
Source: PLoS One. 2024 Nov 14;19(11):e0308742. doi: 10.1371/journal.pone.0308742 (PMC11563381; doi:10.1371/journal.pone.0308742)
Supplement: S1 Table — This table has two separate parts: S1.1 and S1.2. Each has its own notes. (DOCX) [file pone.0308742.s001.docx]

**S1 Table. Sample Descriptions**

**This table has two separate parts: S1.1 and S1.2.**

**S1.1 Table. Exclusion Criteria and Sample Size**

|  | No of countries | No of cities | No of firms |
| --- | --- | --- | --- |
| Raw data | 115 | NA | 111,675 |
| Excluding: cities with <=250K population | 103 | 186 | 37,259 |
| Excluding: war countries | 102 | 181 | 36,901 |
| Excluding: war countries + countries with high GDP | 99 | 176 | 36,547 |
| Excluding: war countries + countries with high GDP + countries with missing GDP data | 98 | 175 | 34,988 |
| Excluding: war countries + countries with high GDP + countries with missing GDP data + gas flare | 98 | 174 | 34,987 |
| Excluding: war countries + countries with high GDP + countries with missing GDP data + gas flare + cities with <20 firms | 98 | 166 | 34,854 |
| Excluding: war countries + countries with high GDP + countries with missing GDP data + gas flare + cities with <20 firms + countries with all innovation data missing | 96 | 164 | 34,690 |

**Notes:**

1. Cities in war countries are: Kabul, Kandahar, Kano, Karachi, Peshawar. These cities locate Afghanistan, Nigeria, Pakistan. Both cities (Kabul and Kandahar) in Afghanistan are in the war zone. For Nigeria and Pakistan, there are cities outside the war zone (like Lagos and Lahore) that are still included in the sample. That is why the number of countries included dropped only by one from 103 to 102 after we exclude war zones.
2. High GDP countries: Israel, Italy, Sweden. Countries with missing GDP: Djibouti, Egypt in 2020. All cities in Egypt have observations in 2013 and 2016 as well. That is why the number of countries only drops by one from 99 to 98 (Djibouti) and number of cities only drops by one from 176 to 175 (Djibouti City) after we exclude observations with missing GDP per capita data.
3. One firm locates in Port Harcourt in Nigeria. The nightlight data there are severely affected by gas flares. We exclude Port Harcourt from the sample.
4. The number of cities is “NA” in the raw data because WBES does not provide information on cities. All city information is derived from external maps described in the “data” section in the paper.
5. 13 cities have fewer than 20 firms in WBES: Sofia in 2019, Bekasi, Seberang Perai, Ecatepec, Benin City, Kazan in 2019, Toamasina, Tianjin, Basrah, Fez in 2013, Krakov in 2013, Samarqand in 2013, Rajshahi. Sofia, Kazan, Fez, Krakov and Samarqand were surveyed in other years. That is why the number of cities drops by 8 after excluding cities with <20 firms.
6. Data on all innovation measures are missing for Antananarivo in Madagascar and Baghdad in Iraq. Therefore, the number of cities drops from 98 to 96 in the last row. Two other cities also have all innovation measures missing: Toamasina in Madagascar and Basrah in Iraq. Both have fewer than 20 firms and are already dropped from the sample in the previous step.

**S1.2 Table. Distribution of the Sample across Country Categories**

| Country categories | Mean | Std |
| --- | --- | --- |
| 1 city selected | 0.304 | 0.46 |
| 2 cities selected | 0.341 | 0.474 |
| 3 cities selected | 0.471 | 0.499 |
| 5 cities selected | 0.363 | 0.481 |
| 6 cities selected | 0.234 | 0.424 |
| 12 cities selected | 0.332 | 0.471 |

**Notes:** for each country category, the table presents the proportion of firms selected in our sample over all firms surveyed by WBES in the country. For example, for countries where 3 cities were selected by us, the mean value refers to the proportion of firms in those 3 cities over all firms surveyed by WBES in the corresponding country. We exclude cities in war areas and high-GDP countries. Cities that have fewer than 20 firms are also excluded from our sample, including: Sofia in 2019, Bekasi, Seberang Perai, Ecatepec, Benin City, Kazan in 2019, Toamasina, Tianjin, Basrah, Fez in 2013, Krakov in 2013, Samarqand in 2013, Rajshahi. Sofia, Kazan, Fez, Krakov and Samarqand.
