# Supplementary material for: Agglomeration costs limit sustainable innovation in cities in developing economies
Source: PLoS One. 2024 Nov 14;19(11):e0308742. doi: 10.1371/journal.pone.0308742 (PMC11563381; doi:10.1371/journal.pone.0308742)
Supplement: S2 Table — The number of firms in each city is obtained by matching firms’ GPS coordinates with city boundaries. Cities that have fewer than 20 firms are excluded from our sample, including: Sofia in 2019, Bekasi, Seberang Perai, Ecatepec, Benin City, Kazan in 2019, Toamasina, Tianjin, Basrah, Fez in 2013, Krakov in 2013, Samarqand in 2013, Rajshahi. Sofia, Kazan, Fez, Krakov and Samarqand. The total number of cities in the list is larger than the total number of distinct cities surveyed in WBES because some cities were surveyed more than once in different years and they were double counted because different firms were surveyed in different years. (DOCX) [file pone.0308742.s002.docx]

**S2 Table. Data Description**

| **country** | city | year | number of firms | nightlight density |
| --- | --- | --- | --- | --- |
| **Albania** | Tirana | 2013 | 130 | 57.965 |
| **Albania** | Tirana | 2019 | 61 | 61.368 |
| **Argentina** | Buenos Aires | 2010 | 445 | 58.917 |
| **Argentina** | Buenos Aires | 2017 | 335 | 62.997 |
| **Argentina** | Cordoba | 2010 | 124 | 47.939 |
| **Argentina** | Cordoba | 2017 | 145 | 54.893 |
| **Armenia** | Erevan | 2013 | 277 | 49.226 |
| **Azerbaijan** | Baku | 2013 | 196 | 30.357 |
| **Azerbaijan** | Baku | 2019 | 161 | 34.410 |
| **Bangladesh** | Chittagong | 2013 | 193 | 51.374 |
| **Bangladesh** | Dhaka | 2013 | 621 | 53.475 |
| **Bangladesh** | Gazipur | 2013 | 34 | 26.212 |
| **Bangladesh** | Khulna | 2013 | 26 | 31.784 |
| **Belarus** | Minsk | 2013 | 114 | 31.375 |
| **Belarus** | Minsk | 2018 | 154 | 22.562 |
| **Benin** | Cotonou | 2016 | 51 | 52.451 |
| **Bolivia** | Santa Cruz de La Sierra | 2010 | 124 | 16.575 |
| **Bolivia** | Santa Cruz de La Sierra | 2017 | 117 | 25.548 |
| **Bosnia and Herzegovina** | Sarajevo | 2013 | 45 | 13.449 |
| **Bosnia and Herzegovina** | Sarajevo | 2019 | 95 | 16.260 |
| **Bulgaria** | Sofia | 2013 | 60 | 27.382 |
| **Burundi** | Bujumbura | 2014 | 88 | 28.109 |
| **Cambodia** | Phnom Penh | 2016 | 126 | 33.684 |
| **Cameroon** | Douala | 2016 | 143 | 18.161 |
| **Cameroon** | Yaounde | 2016 | 147 | 42.101 |
| **Central African Republic** | Bangui | 2011 | 73 | 12.651 |
| **Chad** | NDjamena | 2018 | 138 | 24.895 |
| **Chile** | Santiago | 2010 | 325 | 24.122 |
| **China** | Beijing | 2012 | 87 | 31.489 |
| **China** | Chengdu | 2012 | 80 | 30.841 |
| **China** | Dongguan | 2012 | 34 | 58.521 |
| **China** | Guangzhou | 2012 | 111 | 41.429 |
| **China** | Hangzhou | 2012 | 82 | 30.048 |
| **China** | Nanjing | 2012 | 102 | 29.567 |
| **China** | Shanghai | 2012 | 48 | 52.146 |
| **China** | Shenzhen | 2012 | 121 | 54.665 |
| **China** | Wuhan | 2012 | 87 | 16.910 |
| **Colombia** | Bogota | 2010 | 528 | 20.795 |
| **Colombia** | Bogota | 2017 | 384 | 22.868 |
| **Colombia** | Medellin | 2010 | 172 | 38.755 |
| **Colombia** | Medellin | 2017 | 178 | 39.736 |
| **Colombia** | Santiago de Cali | 2010 | 60 | 31.775 |
| **Colombia** | Santiago de Cali | 2017 | 79 | 31.447 |
| **Congo, Dem. Rep.** | Kinshasa | 2013 | 165 | 2.154 |
| **Congo, Dem. Rep.** | Lubumbashi | 2013 | 54 | 14.602 |
| **Congo, Dem. Rep.** | Mbuji-Mayi | 2013 | 41 | 7.981 |
| **Costa Rica** | San Jose | 2010 | 111 | 59.000 |
| **Croatia** | Zagreb | 2013 | 78 | 47.288 |
| **Croatia** | Zagreb | 2019 | 71 | 46.232 |
| **Czech Republic** | Prague | 2013 | 46 | 57.686 |
| **Czech Republic** | Prague | 2019 | 76 | 56.361 |
| **Côte d'Ivoire** | Abidjan | 2016 | 257 | 45.608 |
| **Dominican Republic** | Santo Domingo | 2016 | 80 | 62.220 |
| **Ecuador** | Guayaquil | 2010 | 106 | 17.041 |
| **Ecuador** | Guayaquil | 2017 | 80 | 21.387 |
| **Egypt** | Alexandria | 2013 | 151 | 29.770 |
| **Egypt** | Alexandria | 2016 | 222 | 34.308 |
| **Egypt** | Cairo | 2013 | 806 | 29.327 |
| **Egypt** | Cairo | 2016 | 483 | 33.883 |
| **Egypt** | Giza | 2013 | 340 | 60.459 |
| **Egypt** | Giza | 2016 | 183 | 61.028 |
| **Egypt** | Port Said | 2013 | 43 | 12.352 |
| **Egypt** | Port Said | 2016 | 40 | 15.509 |
| **Egypt** | Shubra El Kheima | 2013 | 32 | 63.000 |
| **Egypt** | Shubra El Kheima | 2016 | 23 | 63.000 |
| **El Salvador** | San Salvador | 2010 | 148 | 56.286 |
| **El Salvador** | San Salvador | 2016 | 158 | 58.074 |
| **Estonia** | Tallinn | 2013 | 52 | 59.061 |
| **Estonia** | Tallinn | 2019 | 77 | 61.559 |
| **Ethiopia** | Addis Ababa | 2011 | 22 | 30.656 |
| **Ethiopia** | Addis Ababa | 2015 | 452 | 38.385 |
| **Gambia** | Kanifing | 2018 | 69 | 28.720 |
| **Georgia** | Tbilisi | 2013 | 118 | 50.058 |
| **Georgia** | Tbilisi | 2019 | 177 | 52.765 |
| **Ghana** | Accra | 2013 | 285 | 60.665 |
| **Ghana** | Kumasi | 2013 | 91 | 48.226 |
| **Greece** | Athens | 2018 | 84 | 60.400 |
| **Guatemala** | Guatemala City | 2010 | 309 | 50.548 |
| **Guatemala** | Guatemala City | 2017 | 182 | 57.424 |
| **Guinea** | Conakry | 2016 | 87 | 20.529 |
| **Honduras** | Tegucigalpa | 2010 | 153 | 11.894 |
| **Honduras** | Tegucigalpa | 2016 | 158 | 16.373 |
| **Hungary** | Budapest | 2013 | 104 | 60.740 |
| **Hungary** | Budapest | 2019 | 73 | 58.648 |
| **India** | Ahmadabad | 2014 | 130 | 62.090 |
| **India** | Bangalore | 2014 | 567 | 43.765 |
| **India** | Chennai | 2014 | 275 | 58.892 |
| **India** | Delhi | 2014 | 990 | 44.058 |
| **India** | Hyderabad | 2014 | 269 | 27.805 |
| **India** | Jaipur | 2014 | 179 | 33.358 |
| **India** | Kanpur | 2014 | 29 | 20.337 |
| **India** | Kolkata | 2014 | 261 | 63.000 |
| **India** | Lucknow | 2014 | 38 | 34.389 |
| **India** | Mumbai | 2014 | 364 | 58.203 |
| **India** | Pune | 2014 | 58 | 26.536 |
| **India** | Surat | 2014 | 64 | 39.897 |
| **Indonesia** | Bandung | 2015 | 35 | 55.759 |
| **Indonesia** | Jakarta | 2015 | 173 | 61.693 |
| **Indonesia** | Medan | 2015 | 53 | 55.154 |
| **Indonesia** | Surabaya | 2015 | 65 | 58.340 |
| **Indonesia** | Tangerang | 2015 | 47 | 60.523 |
| **Jordan** | Amman | 2013 | 107 | 58.120 |
| **Jordan** | Amman | 2019 | 57 | 60.889 |
| **Kazakhstan** | Almaty | 2013 | 50 | 57.815 |
| **Kazakhstan** | Almaty | 2019 | 171 | 60.810 |
| **Kenya** | Kisumu | 2013 | 86 | 5.909 |
| **Kenya** | Kisumu | 2018 | 70 | 13.701 |
| **Kenya** | Mombasa | 2013 | 118 | 33.651 |
| **Kenya** | Mombasa | 2018 | 70 | 39.106 |
| **Kenya** | Nairobi | 2013 | 343 | 35.742 |
| **Kenya** | Nairobi | 2018 | 329 | 50.062 |
| **Kyrgyz Republic** | Bishkek | 2013 | 114 | 52.958 |
| **Kyrgyz Republic** | Bishkek | 2019 | 147 | 54.154 |
| **Lao PDR** | Vientiane | 2016 | 135 | 10.257 |
| **Lao PDR** | Vientiane | 2018 | 130 | 11.235 |
| **Latvia** | Riga | 2013 | 157 | 17.542 |
| **Latvia** | Riga | 2019 | 103 | 19.248 |
| **Lebanon** | Beirut | 2013 | 94 | 62.607 |
| **Lebanon** | Beirut | 2019 | 49 | 63.000 |
| **Liberia** | Monrovia | 2017 | 46 | 15.469 |
| **Lithuania** | Vilniaus | 2013 | 72 | 13.715 |
| **Lithuania** | Vilniaus | 2019 | 96 | 15.319 |
| **Malawi** | Lilongwe City | 2014 | 185 | 22.724 |
| **Malaysia** | Kuala Lumpur | 2015 | 29 | 62.997 |
| **Mali** | Bamako | 2016 | 122 | 50.695 |
| **Mauritania** | Nouakchott | 2014 | 89 | 8.133 |
| **Mexico** | Leon | 2010 | 163 | 30.262 |
| **Mexico** | Mexico City | 2010 | 244 | 39.065 |
| **Mexico** | Puebla | 2010 | 23 | 26.772 |
| **Mongolia** | Ulan Bator | 2013 | 201 | 7.487 |
| **Mongolia** | Ulan Bator | 2019 | 149 | 9.330 |
| **Morocco** | Casablanca | 2013 | 128 | 41.145 |
| **Morocco** | Casablanca | 2019 | 131 | 51.549 |
| **Morocco** | Fez | 2019 | 71 | 19.945 |
| **Mozambique** | Maputo | 2018 | 200 | 38.107 |
| **Mozambique** | Nampula | 2018 | 67 | 6.669 |
| **Myanmar** | Mandalay | 2014 | 124 | 6.454 |
| **Myanmar** | Mandalay | 2016 | 133 | 10.426 |
| **Myanmar** | Yangon | 2014 | 259 | 20.115 |
| **Myanmar** | Yangon | 2016 | 225 | 21.152 |
| **Namibia** | Windhoek | 2014 | 210 | 15.546 |
| **Nepal** | Kathmandu | 2013 | 150 | 53.339 |
| **Nepal** | Pokhara | 2013 | 33 | 11.813 |
| **Nicaragua** | Managua | 2010 | 144 | 24.850 |
| **Nicaragua** | Managua | 2016 | 164 | 28.299 |
| **Niger** | Niamey | 2017 | 126 | 26.295 |
| **Nigeria** | Ibadan | 2014 | 110 | 9.903 |
| **Nigeria** | Kaduna | 2014 | 65 | 47.471 |
| **Nigeria** | Lagos | 2014 | 254 | 42.686 |
| **North Macedonia** | Skopje | 2013 | 114 | 38.667 |
| **North Macedonia** | Skopje | 2019 | 97 | 40.908 |
| **Pakistan** | Faisalabad | 2013 | 34 | 15.540 |
| **Pakistan** | Gujranwala | 2013 | 65 | 12.386 |
| **Pakistan** | Islamabad-Rawalpindi | 2013 | 20 | 16.627 |
| **Pakistan** | Lahore | 2013 | 44 | 37.565 |
| **Panama** | Panama City | 2010 | 167 | 12.487 |
| **Paraguay** | Asuncion | 2010 | 180 | 56.089 |
| **Paraguay** | Asuncion | 2017 | 172 | 62.339 |
| **Peru** | Arequipa | 2010 | 122 | 21.299 |
| **Peru** | Arequipa | 2017 | 161 | 27.843 |
| **Peru** | Lima | 2010 | 646 | 27.165 |
| **Peru** | Lima | 2017 | 479 | 32.812 |
| **Philippines** | Cebu City | 2015 | 55 | 22.281 |
| **Philippines** | Manila | 2015 | 508 | 58.535 |
| **Poland** | Krakov | 2019 | 39 | 57.694 |
| **Poland** | Warsaw | 2013 | 53 | 61.147 |
| **Poland** | Warsaw | 2019 | 88 | 61.013 |
| **Portugal** | Lisbon | 2019 | 25 | 63.000 |
| **Romania** | Bucharest | 2013 | 90 | 62.446 |
| **Romania** | Bucharest | 2019 | 102 | 62.354 |
| **Russia** | City of St. Petersburg | 2012 | 125 | 46.351 |
| **Russia** | City of St. Petersburg | 2019 | 156 | 60.760 |
| **Russia** | Kazan | 2012 | 69 | 16.323 |
| **Russia** | Moscow | 2012 | 175 | 37.628 |
| **Russia** | Moscow | 2019 | 178 | 47.769 |
| **Russia** | Novosibirsk | 2012 | 114 | 24.509 |
| **Russia** | Novosibirsk | 2019 | 65 | 29.539 |
| **Russia** | Yekaterinburg | 2012 | 100 | 38.214 |
| **Russia** | Yekaterinburg | 2019 | 121 | 44.649 |
| **Rwanda** | Kigali | 2011 | 232 | 9.581 |
| **Senegal** | Dakar | 2014 | 183 | 60.563 |
| **Serbia** | Belgrade | 2013 | 116 | 27.844 |
| **Serbia** | Belgrade | 2019 | 118 | 31.023 |
| **Sierra Leone** | Freetown | 2017 | 53 | 22.423 |
| **Slovak Republic** | Bratislava | 2013 | 62 | 46.047 |
| **Slovak Republic** | Bratislava | 2019 | 94 | 47.500 |
| **Slovenia** | Ljubljana | 2013 | 49 | 36.998 |
| **Slovenia** | Ljubljana | 2019 | 58 | 35.769 |
| **South Sudan** | Juba | 2014 | 402 | 0.267 |
| **Sudan** | Khartoum | 2014 | 159 | 58.091 |
| **Sudan** | Omdurman | 2014 | 101 | 14.156 |
| **Tajikistan** | Dushanbe | 2013 | 90 | 49.298 |
| **Tajikistan** | Dushanbe | 2019 | 98 | 54.302 |
| **Tanzania** | Arusha | 2013 | 85 | 10.424 |
| **Tanzania** | Dar es Salaam | 2013 | 362 | 16.802 |
| **Tanzania** | Mwanza | 2013 | 45 | 8.706 |
| **Thailand** | Bangkok | 2016 | 267 | 54.714 |
| **Togo** | Lome | 2016 | 95 | 38.398 |
| **Tunisia** | Tunis | 2013 | 128 | 55.293 |
| **Turkey** | Ankara | 2013 | 129 | 35.431 |
| **Turkey** | Ankara | 2019 | 102 | 38.070 |
| **Turkey** | Istanbul | 2013 | 259 | 28.868 |
| **Turkey** | Istanbul | 2019 | 129 | 35.258 |
| **Turkey** | Izmir | 2013 | 130 | 37.688 |
| **Turkey** | Izmir | 2019 | 55 | 44.607 |
| **Uganda** | Kampala | 2013 | 317 | 51.557 |
| **Uganda** | Kira | 2013 | 64 | 24.398 |
| **Ukraine** | Kharkiv | 2013 | 50 | 56.902 |
| **Ukraine** | Kharkiv | 2019 | 59 | 59.135 |
| **Ukraine** | Kyiv | 2013 | 173 | 50.542 |
| **Ukraine** | Kyiv | 2019 | 212 | 53.198 |
| **Uruguay** | Montevideo | 2010 | 419 | 45.123 |
| **Uruguay** | Montevideo | 2017 | 192 | 52.422 |
| **Uzbekistan** | Samarqand | 2019 | 39 | 16.384 |
| **Uzbekistan** | Tashkent | 2013 | 129 | 58.381 |
| **Uzbekistan** | Tashkent | 2019 | 177 | 59.411 |
| **Venezuela** | Caracas | 2010 | 22 | 1.168 |
| **Vietnam** | Da Nang | 2015 | 68 | 40.533 |
| **Vietnam** | Hanoi | 2015 | 113 | 46.296 |
| **Vietnam** | Ho Chi Minh City | 2015 | 156 | 30.936 |
| **West Bank and Gaza** | Gaza | 2013 | 58 | 43.800 |
| **West Bank and Gaza** | Gaza | 2019 | 70 | 50.558 |
| **Yemen** | Aden | 2010 | 57 | 41.011 |
| **Yemen** | Aden | 2013 | 29 | 39.511 |
| **Yemen** | Sanaa | 2010 | 114 | 33.857 |
| **Yemen** | Sanaa | 2013 | 131 | 34.312 |
| **Zambia** | Lusaka | 2013 | 326 | 42.773 |
| **Zambia** | Lusaka | 2019 | 285 | 51.075 |
| **Zimbabwe** | Harare | 2011 | 304 | 32.322 |
| **Zimbabwe** | Harare | 2016 | 234 | 33.027 |
