# Supplementary material for: Agglomeration costs limit sustainable innovation in cities in developing economies
Source: PLoS One. 2024 Nov 14;19(11):e0308742. doi: 10.1371/journal.pone.0308742 (PMC11563381; doi:10.1371/journal.pone.0308742)
Supplement: S3 Table — The table reports the ordered logit regression for the innovation index. We control for per capita GDP in each country and include geographic region and year fixed effects. The key independent variables are the interaction between city population and nightlight density, and the interaction between city population and the quadratic term of nightlight density. P-values are in parentheses, and 95% confidence intervals are in square brackets below p-values. Ordered Logit estimates do not include a constant. NTL and GDP are lagged. City population data comes from the most recent census data in each country. *** p<0.01, ** p<0.05, * p<0.1. (DOCX) [file pone.0308742.s003.docx]

**S3 Table. Regressions for Fig 2: Moderating Effects of City Population Size**

|  | (1) |
| --- | --- |
| VARIABLES | Innovation Index |
|  |  |
| Ln(Night Light) | 0.098*** |
|  | (0.008) |
|  | [0.026,0.170] |
| Ln(Night Light) Sqr | -0.010 |
|  | (0.231) |
|  | [-0.027,0.007] |
| City Population | -0.228*** |
|  | (0.000) |
|  | [-0.298,-0.158] |
| Ln(Night Light)*City Population | 0.164*** |
|  | (0.000) |
|  | [0.122,0.207] |
| Ln(Night Light) Sqr*City Population | -0.027*** |
|  | (0.000) |
|  | [-0.034,-0.020] |
| Per Capita GDP | 0.051 |
|  | (0.147) |
|  | [-0.018,0.121] |
|  |  |
| Observations | 31,798 |
| Conflict Regions | Exclude |
| GDPpc>30K Countries | Exclude |
| Region Fixed Effects | Yes |
| Year Fixed Effects | Yes |
| Pseudo R2 | 0.0746 |
