# Supplementary material for: Agglomeration costs limit sustainable innovation in cities in developing economies
Source: PLoS One. 2024 Nov 14;19(11):e0308742. doi: 10.1371/journal.pone.0308742 (PMC11563381; doi:10.1371/journal.pone.0308742)
Supplement: S4 Table — The table reports ordered logit regression for the innovation index for large cities (>5M population) in column 1 and small cities (<5M population) in column 2. The key independent variables are the nightlight density and its quadratic term. We control for per capita GDP in each country and include geographic region and year fixed effects. P-values are in parentheses, and 95% confidence intervals are in square brackets below p-values. Ordered Logit estimates do not include a constant. NTL and GDP are lagged. Coefficients can be interpreted as the increase in the log odds of being in a higher innovation level versus all lower innovation levels after a 1% increase in nightlight density. *** p<0.01, ** p<0.05, * p<0.1. (DOCX) [file pone.0308742.s004.docx]

**S4 Table. Regressions for Fig 3: Split Sample for Large and Small Cities**

|  | (1) | (2) |
| --- | --- | --- |
| VARIABLES | Large Cities (>5M) | Small Cities (<=5M) |
|  |  |  |
| Ln(Night Light) | 1.149*** | 0.178*** |
|  | (0.000) | (0.000) |
|  | [0.713,1.584] | [0.111,0.244] |
| Ln(Night Light) Sqr | -0.245*** | -0.025*** |
|  | (0.000) | (0.001) |
|  | [-0.320,-0.169] | [-0.040,-0.010] |
| Per Capita GDP | -0.209 | 0.169*** |
|  | (0.298) | (0.000) |
|  | [-0.602,0.185] | [0.099,0.240] |
|  |  |  |
| Observations | 11,215 | 20,583 |
| Conflict Regions | Exclude | Exclude |
| GDPpc>30K Countries | Exclude | Exclude |
| Region Fixed Effects | Yes | Yes |
| Year Fixed Effects | Yes | Yes |
