# Supplementary material for: Agglomeration costs limit sustainable innovation in cities in developing economies
Source: PLoS One. 2024 Nov 14;19(11):e0308742. doi: 10.1371/journal.pone.0308742 (PMC11563381; doi:10.1371/journal.pone.0308742)
Supplement: S5 Table — The table reports OLS regression results for all previous results based on ordered logit models. Columns 1–4 report OLS results for the corresponding columns in Table 2. Column 5 relates to the model in Table in S3 Table. Columns 6 and 7 report the corresponding OLS results based on ordered logit models in Table in S4 Table. We control for per capita GDP in each country and include geographic region and year fixed effects. P-values are in parentheses, and 95% confidence intervals are in square brackets below p-values. NTL and GDP are lagged. *** p<0.01, ** p<0.05, * p<0.1. (DOCX) [file pone.0308742.s005.docx]

**S5 Table. Robustness Check: Using OLS Regressions**

|  | (1) | (2) | (3) | (4) | (5) | (6) | (7) |
| --- | --- | --- | --- | --- | --- | --- | --- |
| VARIABLES | NewPro | NewEst | RND | Innovation index | Interact with City Population | Large Cities | Small Cities |
|  |  |  |  |  |  |  |  |
| Ln(Night Light) | 0.037*** | 0.081*** | 0.030*** | 0.149*** | 0.062*** | 0.750*** | 0.107*** |
|  | (0.000) | (0.000) | (0.000) | (0.000) | (0.003) | (0.000) | (0.000) |
|  | [0.021,0.053] | [0.065,0.096] | [0.016,0.044] | [0.115,0.184] | [0.021,0.102] | [0.521,0.979] | [0.071,0.144] |
| Ln(Night Light) Sqr | -0.005*** | -0.015*** | -0.003** | -0.024*** | -0.006 | -0.164*** | -0.015*** |
|  | (0.003) | (0.000) | (0.023) | (0.000) | (0.191) | (0.000) | (0.000) |
|  | [-0.009,-0.002] | [-0.018,-0.012] | [-0.006,-0.000] | [-0.031,-0.017] | [-0.015,0.003] | [-0.204,-0.124] | [-0.023,-0.007] |
| City Population |  |  |  |  | -0.113*** |  |  |
|  |  |  |  |  | (0.000) |  |  |
|  |  |  |  |  | [-0.149,-0.077] |  |  |
| Ln(Night Light)*City Population |  |  |  |  | 0.087*** |  |  |
|  |  |  |  |  | (0.000) |  |  |
|  |  |  |  |  | [0.065,0.109] |  |  |
| Ln(Night Light) Sqr*City Population |  |  |  |  | -0.015*** |  |  |
|  |  |  |  |  | (0.000) |  |  |
|  |  |  |  |  | [-0.018,-0.011] |  |  |
| Per Capita GDP | -0.009 | 0.006 | 0.039*** | 0.036** | 0.037** | 0.261** | 0.090*** |
|  | (0.258) | (0.434) | (0.000) | (0.046) | (0.042) | (0.011) | (0.000) |
|  | [-0.026,0.007] | [-0.010,0.022] | [0.025,0.053] | [0.001,0.071] | [0.001,0.072] | [0.060,0.463] | [0.055,0.126] |
| Constant | 0.221*** | 0.243*** | 0.298*** | 0.757*** | 0.799*** | 0.579*** | 0.702*** |
|  | (0.000) | (0.000) | (0.000) | (0.000) | (0.000) | (0.002) | (0.000) |
|  | [0.171,0.272] | [0.194,0.291] | [0.255,0.341] | [0.649,0.864] | [0.686,0.913] | [0.206,0.952] | [0.583,0.820] |
|  |  |  |  |  |  |  |  |
| Observations | 32,675 | 32,095 | 32,171 | 31,798 | 31,798 | 11,215 | 20,583 |
| R-squared | 0.10 | 0.16 | 0.09 | 0.17 | 0.17 | 0.25 | 0.15 |
| Conflict Regions | Exclude | Exclude | Exclude | Exclude | Exclude | Exclude | Exclude |
| GDPpc>30K Countries | Exclude | Exclude | Exclude | Exclude | Exclude | Exclude | Exclude |
| Region Fixed Effects | Yes | Yes | Yes | Yes | Yes | Yes | Yes |
| Year Fixed Effects | Yes | Yes | Yes | Yes | Yes | Yes | Yes |
