# Supplementary material for: Agglomeration costs limit sustainable innovation in cities in developing economies
Source: PLoS One. 2024 Nov 14;19(11):e0308742. doi: 10.1371/journal.pone.0308742 (PMC11563381; doi:10.1371/journal.pone.0308742)
Supplement: S6 Table — The table reports, with firm level controls, logit regressions for each individual item in the innovation index in columns 1–3, and the ordered logit regression for the innovation index in column 4. The key independent variables are the logarithms of nightlight density and its quadratic term. We control for per capita GDP in each country and include geographic region and year fixed effects. We additionally control for firm-level controls, i.e., age, firm size dummies indicating whether a firm is medium-sized (20–99 employees) and whether a firm is large-sized (100 and over 100 employees). P-values are in parentheses, and 95% confidence intervals are in square brackets below p-values. Ordered Logit estimates do not include a constant. Number of observations varies because of missing values for each measure. NTL and GDP are lagged. Coefficients can be interpreted as the increase in the log odds of being in a higher innovation level versus all lower innovation levels after a 1% increase in nightlight density. *** p<0.01, ** p<0.05, * p<0.1. (DOCX) [file pone.0308742.s006.docx]

**S6 Table. Robustness Tests: Adding Firm-Level Controls**

|  | (1) | (2) | (3) | (4) |
| --- | --- | --- | --- | --- |
| VARIABLES | NewPro | NewEst | RND | Innovation Index |
|  |  |  |  |  |
| Ln(Night Light) | 0.119*** | 0.319*** | 0.096** | 0.201*** |
|  | (0.001) | (0.000) | (0.033) | (0.000) |
|  | [0.048,0.189] | [0.246,0.391] | [0.008,0.185] | [0.140,0.263] |
| Ln(Night Light) Sqr | -0.020** | -0.062*** | -0.016* | -0.037*** |
|  | (0.011) | (0.000) | (0.088) | (0.000) |
|  | [-0.035,-0.005] | [-0.078,-0.047] | [-0.035,0.002] | [-0.051,-0.024] |
| Age | 0.003*** | 0.001 | 0.004*** | 0.003*** |
|  | (0.000) | (0.491) | (0.000) | (0.000) |
|  | [0.002,0.005] | [-0.001,0.002] | [0.002,0.006] | [0.002,0.005] |
| Firm Size Medium | 0.374*** | 0.454*** | 0.681*** | 0.508*** |
|  | (0.000) | (0.000) | (0.000) | (0.000) |
|  | [0.320,0.429] | [0.396,0.512] | [0.612,0.750] | [0.459,0.557] |
| Firm Size Large | 0.726*** | 0.872*** | 1.277*** | 1.024*** |
|  | (0.000) | (0.000) | (0.000) | (0.000) |
|  | [0.661,0.790] | [0.804,0.941] | [1.201,1.353] | [0.966,1.083] |
| Per Capita GDP | -0.074* | -0.009 | 0.267*** | 0.026 |
|  | (0.065) | (0.838) | (0.000) | (0.464) |
|  | [-0.152,0.005] | [-0.097,0.078] | [0.168,0.366] | [-0.044,0.096] |
| Constant | -1.599*** | -1.738*** | -1.947*** |  |
|  | (0.000) | (0.000) | (0.000) |  |
|  | [-1.827,-1.371] | [-1.980,-1.496] | [-2.237,-1.657] |  |
|  |  |  |  |  |
| Observations | 32,243 | 31,694 | 31,764 | 31,419 |
| R-squared | NA | NA | NA | NA |
| Method | Logit | Logit | Logit | Logit |
| Conflict | Exclude | Exclude | Exclude | Exclude |
| GDPpc>30000 | Exclude | Exclude | Exclude | Exclude |
| Region FE | Yes | Yes | Yes | Yes |
| Year FE | Yes | Yes | Yes | Yes |
| Pseudo R2 | 0.0940 | 0.149 | 0.122 | 0.0919 |
