# Supplementary material for: Agglomeration costs limit sustainable innovation in cities in developing economies
Source: PLoS One. 2024 Nov 14;19(11):e0308742. doi: 10.1371/journal.pone.0308742 (PMC11563381; doi:10.1371/journal.pone.0308742)
Supplement: S7 Table — The table reports generalized ordered logit regressions which relax the proportional odds assumption of standard ordered logit regressions. Columns 1, 2 and 3 present the coefficients for the likelihood of: having zero versus all other categories of innovation; having zero or low innovation versus medium and high innovation; having zero, low and medium innovation versus high innovation. The key independent variables are night light density and its quadratic term. We control for per capita GDP in each country and include geographic region and year fixed effects. P-values are in parentheses, and 95% confidence intervals are in square brackets below p-values. Number of observations varies because of missing values for each measure. NTL and GDP are lagged. *** p<0.01, ** p<0.05, * p<0.1. (DOCX) [file pone.0308742.s007.docx]

**S7 Table. Generalised Ordered Logit Model: Testing the Assumption of Ordered Logit Model**

|  | (1) | (2) | (3) |
| --- | --- | --- | --- |
| VARIABLES | Zero vs. low, medium, high | Zero, low vs. medium, high | Zero, low, medium vs. high |
|  |  |  |  |
| Ln(Night Light) | 0.12*** | 0.39*** | 0.27*** |
|  | (0.00) | (0.00) | (0.00) |
|  | [0.05,0.20] | [0.31,0.46] | [0.16,0.38] |
| Ln(Night Light) Sqr | -0.02*** | -0.07*** | -0.03** |
|  | (0.00) | (0.00) | (0.02) |
|  | [-0.04,-0.01] | [-0.08,-0.05] | [-0.05,-0.00] |
| Per Capita GDP | 0.00 | 0.00** | 0.00 |
|  | (0.69) | (0.04) | (0.10) |
|  | [-0.00,0.00] | [0.00,0.00] | [-0.00,0.00] |
| Constant | -0.39*** | -1.68*** | -2.59*** |
|  | (0.00) | (0.00) | (0.00) |
|  | [-0.62,-0.15] | [-1.93,-1.44] | [-2.99,-2.19] |
|  |  |  |  |
| Observations | 31,798 | 31,798 | 31,798 |
| Method | Generalized Ordered Logit | Generalized Ordered Logit | Generalized Ordered Logit |
| Conflict Regions | Exclude | Exclude | Exclude |
| GDPpc>30000 | Exclude | Exclude | Exclude |
| Region Fixed Effects | Yes | Yes | Yes |
| Year Fixed Effects | Yes | Yes | Yes |
| Pseudo R2 | 0.0804 | 0.0804 | 0.0804 |
