# Supplementary material for: Agglomeration costs limit sustainable innovation in cities in developing economies
Source: PLoS One. 2024 Nov 14;19(11):e0308742. doi: 10.1371/journal.pone.0308742 (PMC11563381; doi:10.1371/journal.pone.0308742)
Supplement: S8 Table — Data comes from the Urban Indicators Database published by the UN-Habitat (https://data.unhabitat.org/pages/datasets), which covers information on both urban population and urban transport in cities in developing countries as well as developed countries. We first identify large cities as those with more than 5 million urban population in 2015, consistent with our main analysis, which leads to 45 cities in developing countries and 14 in developed countries. The Urban Indicators Database provides information on the proportion of urban population that has convenient access to public transport, defined as the estimated share of urban population with access to a public transport stop within a walking distance of 500 meters (for low-capacity public transport systems) and/or 1000 meters (for high-capacity public transport systems). We perform a one-way t-test to compare the mean value of the proportion of urban population that has convenient access to public transport between 45 cities in developing countries and 14 cities in developed countries. The table shows the mean values, standard deviations and confidence intervals for the two groups, and the t-statistics and p-value for the difference in their mean values. (DOCX) [file pone.0308742.s008.docx]

**S8 Table. Urban Transport Infrastructure in Large Cities in Developed and Developing Countries: T-test Results.**

|  | Obs | Mean Value | Standard Deviation | 95% Confidence Interval |
| --- | --- | --- | --- | --- |
| Cities in developing countries | 45 | 59.741 | 24.966 | [52.241, 67.242] |
| Cities in developed countries | 14 | 72.932 | 23.485 | [59.372, 86.492] |
|  |  |  |  |  |
| *Mean Value Difference* | *-13.191* | | | |
| *T-statistics* | *-1.75* | | | |
| *P-value* | *0.043* | | | |
