# Supplementary figures and images for: Agglomeration costs limit sustainable innovation in cities in developing economies
Source: PLoS One. 2024 Nov 14;19(11):e0308742. doi: 10.1371/journal.pone.0308742 (PMC11563381; doi:10.1371/journal.pone.0308742)

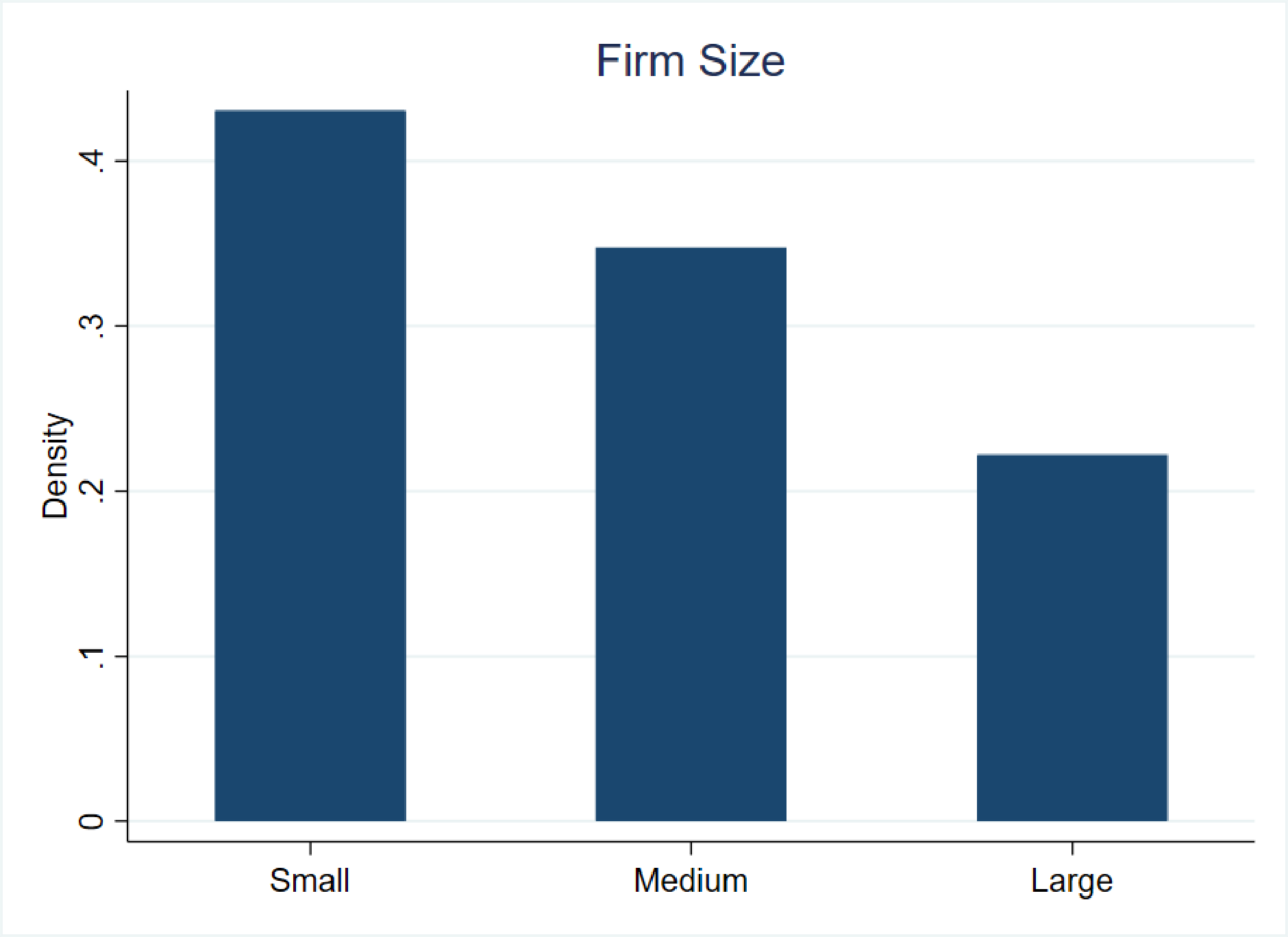

Supplement: S1 Fig — The Figure shows the distribution of firm sizes in our sample. The WBES surveys distinguish firms into small firms (5–19 employees), medium firms (20–99 employees), and large firms (100+ employees). The figure shows that in our sample, 43% of firms are small, 35% of firms are medium firms and 22% are large firms, suggesting that our sample is representative with respect to firm sizes. (TIF) [file pone.0308742.s009.tif]

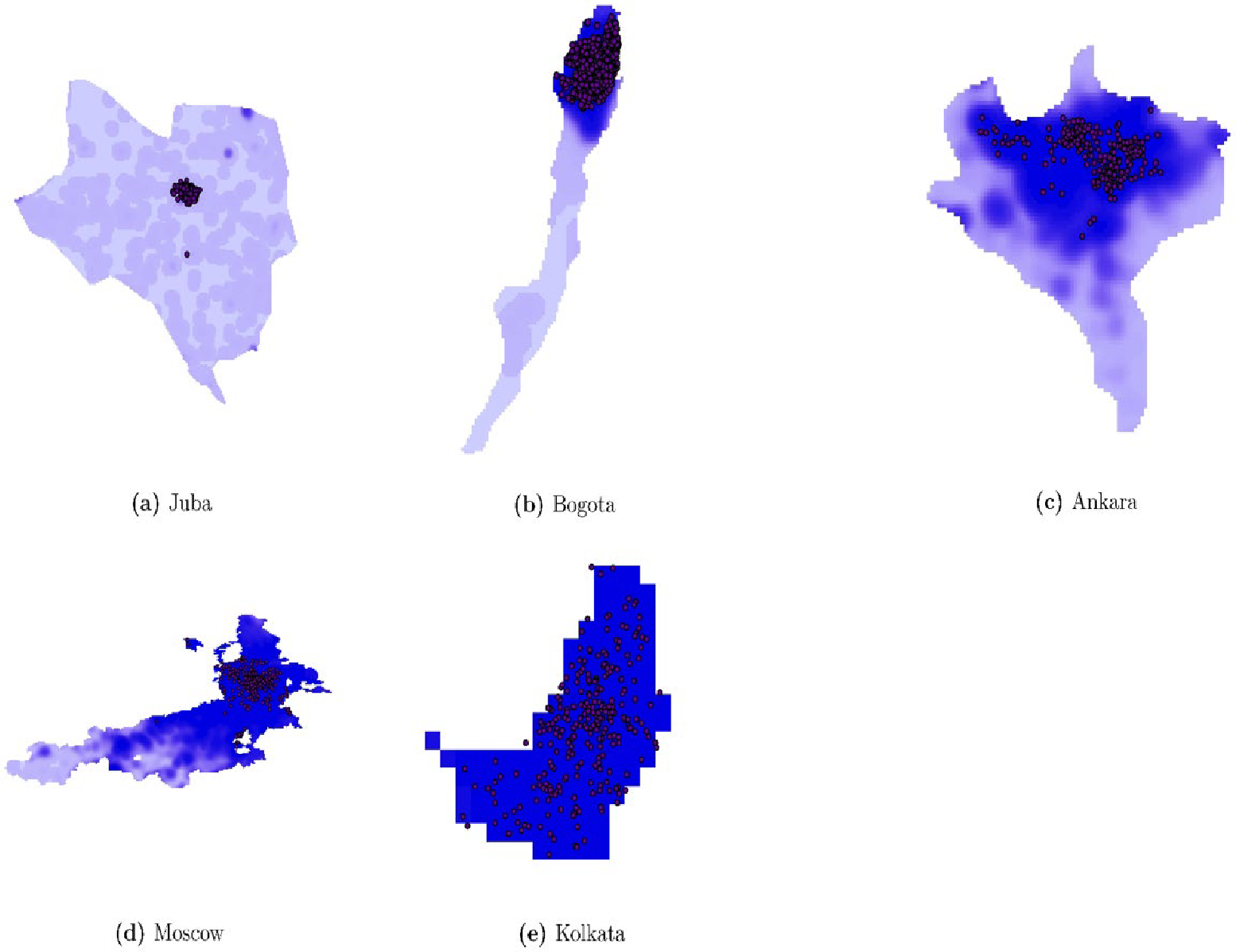

Supplement: S2 Fig — The Figure shows the distribution of firms in five cities ranked from the lowest through the median to among the highest light density: Juba (South Sudan); Bogota (Columbia); Ankara (Turkey); Moscow (Russia) and Kolkata (India). The respective night light densities (ascending rank order in parentheses) are 0.3 (1); 22.9 (58); 38.1(114); 47.8 (148); and 63 (228). For each city, the number of firms shown in the Figure are 402; 912; 231; 353; and 261. The number 228 is larger than the total number of cities because some cities were surveyed more than once in different years and are double counted because different firms were surveyed in different years. Night light data is based on the harmonised version derived by Li et al. (2020) because different satellites were used in 1992–2013 (Defense Meteorological Satellite Program (DMSP)) and 2012–2018 (Visible Infrared Imaging Radiometer Suite (VIIRS) from the Suomi satellite). Darker colour indicates higher night light luminosity. Maps indicating the boundaries of urban and suburban administrative subdivisions in each city are obtained from the Database of Global Administrative Areas (https://gadm.org/download_country_v3.html). The Pearson correlation between the city rank by luminosity and GDP per capita is 0.24, which partly confirms that our NTL measure is a good proxy for social and economic activities. Each dot refers to a firm. The location of the firm is based on the GPS coordinate in WBES. Source: own calculations. (TIF) [file pone.0308742.s010.tif]
