# Supplementary material for: Agglomeration costs limit sustainable innovation in cities in developing economies
Source: PLoS One. 2024 Nov 14;19(11):e0308742. doi: 10.1371/journal.pone.0308742 (PMC11563381; doi:10.1371/journal.pone.0308742)
Supplement: S1 File — (DOCX) [file pone.0308742.s011.docx]

**S1 File. Testing the assumption of the Ordered Logit Model**

1. Ordered logit model and individual firms’ decision making:

The ordered logit regression directly links to our agent-based view of innovation by modeling individual firms’ decision making. Suppose the latent innovation activity $Y_{ij}^{*}$ of firm *i* in city *j* is associated with the density of economic activities in the city proxied by nightlight $N_{j}$. We consider both benefits and costs of agglomeration and therefore include both linear and quadratic terms of nightlight in our model specification:

$Y_{ij}^{*}=\alpha+\beta_{1}\ln N_{j}+\beta_{2}\left( \ln N_{j} \right)^{2}$

We cannot directly observe $Y_{ij}^{*}$ because measures based on patent data or flow of knowledge are either not universally available or not appropriate in developing countries. We therefore construct an innovation index $Y_{ij}$ which is an increasing function of $Y_{ij}^{*}$ to capture firms’ capability of innovation. Firm *i* decides to have zero, low, medium or high levels of innovation if $Y_{ij}$ equals 0, 1, 2 or 3. Suppose M is the total number of categories of the ordinal dependent variable and *m*=1, 2, …, M. A random factor $\epsilon_{ij}$ follows a logistic distribution. Firm *i* chooses to have an innovation level *m* if:

$$\Pr\left( Y_{ij}=m \right)=\Pr\left( \kappa_{m-1}<Y_{ij}^{*}+\epsilon_{ij}\leq\kappa_{m} \right)$$

$\kappa_{m}$ is the cutoff point where $\kappa_{0}=-\infty$ and $\kappa_{M}=+\infty$.

The ordered logit model is specified as:

$$\Pr\left( Y_{ij}>m \right)=\frac{exp\left( \alpha_{m}+\beta_{1}\ln N_{j}+\beta_{2}\left( lnN_{j} \right)^{2} \right)}{1+\{exp\left( \alpha_{m}+\beta_{1}\ln N_{j}+\beta_{2}\left( lnN_{j} \right)^{2} \right)\}}$$

We expect that $\beta_{2}<0$ and $\beta_{1}>0$.

2. Testing the assumption of the ordered logit model:

The key assumption for the ordered logit model is the *proportional odds assumption*, which is also called as *parallel regressions assumption* or *parallel-lines assumption*. That is, $\beta_{1}$ and $\beta_{2}$ should be the same for each value of *m*. In other words, the coefficients for night light density that describe the relationship between no innovation versus all other innovation levels should be the same as the coefficients that describe the relationship between high innovation versus all other innovation levels.

Literature recommends not to use formal tests such as Brant tests to verify the proportional odds assumption, because even substantively trivial violations of the assumption can become statistically significant in a very large sample which is the case for this study[1,2]. Instead, one can use results from generalized ordered logit models that relax the proportional odds assumption as a benchmark to decide if the deviations from the proportional odds assumption are substantively important. For example, coefficients that differ in both magnitude and direction across different values of *m* can be considered as important violations of the assumption. We follow this approach by estimating a generalized ordered logit model which allows $\beta_{1}$ and $\beta_{2}$ to differ between different values of *m* and report the results in Table in S7 Table. Columns 1, 2 and 3 report the coefficients for zero innovation versus other levels; zero and low innovation versus other levels; zero, low and medium innovation versus high innovation, respectively. If the proportional odds assumption is not violated, coefficients for the same independent variable in all three columns should not be significantly different – at least the signs should be consistent. In Table in S7 Table, $\beta_{1}$ are all positive and significant across all three columns (b=0.12, 0.37 and 0.27 in columns 1, 2 and 3), while $\beta_{2}$ are all negative and significant across all three columns with similar magnitude (b=-0.02, -0.07 and -0.03 in columns 1, 2 and 3). These results suggest there is no strong evidence that the proportional odds assumption of the ordered logit mode is violated.

**Reference**

1. Williams R. Understanding and interpreting generalized ordered logit models. J Math Sociol. 2016;40: 7–20.

2. Fullerton A, Xu J. Ordered regression models: Parallel, partial, and non-parallel alternatives. Boca Raton: CRC Press; 2016.
