# Supplementary material for: Agglomeration costs limit sustainable innovation in cities in developing economies
Source: PLoS One. 2024 Nov 14;19(11):e0308742. doi: 10.1371/journal.pone.0308742 (PMC11563381; doi:10.1371/journal.pone.0308742)
Supplement: Attachment — Submitted filename: Response letter.docx [file pone.0308742.s012.docx]

**Response Letters**

We first provide our response to the two reviewers. We then present our response to the journal office regarding journal requirements.

Response to the reviewers

Reviewer #1: The paper "Urban Agglomeration and Innovation in Developing Economies" presents two significant contributions to the field of urban economics and innovation studies. Firstly, the authors introduce a novel framework that challenges the conventional understanding of the relationship between urban agglomeration and innovation in developing countries. This framework suggests that urban agglomeration may not lead to sustainable improvements in innovation within these contexts, contrary to prevailing assumptions. This conceptual shift represents a significant contribution to the discourse on urban development and innovation in developing economies. Secondly, the paper addresses the methodological challenges faced by empirical studies on a global scale, particularly in developing countries. The authors highlight the difficulty of obtaining comprehensive and consistently defined measures, as well as the unreliable collection of data related to urban agglomeration and innovation. To overcome these challenges, the authors leverage recent advancements in data collection techniques to create a unique database that measures both variables consistently across a large sample of developing countries. This methodological approach represents a valuable contribution to the field, providing a framework for future research in similar contexts.
Here are two advices:
1. While the paper highlights the importance of the dataset, there are areas where the presentation could be improved. The authors extensively discuss the selection process of cities in the dataset, but they could provide more innovative insights into how the new data is integrated. It would be beneficial for the paper to clearly demonstrate how the dataset is reconfigured or reorganized, rather than simply being used in its raw form. Strengthening this aspect of the paper would enhance its contribution to the field.

**Response:** *In response to your comment, we revised the paper to further emphasize and clarify how we contribute to empirical studies on urban agglomeration and innovation in developing countries by integrating the two datasets (nightlights and the World Bank Enterprise Survey). When explaining our second contribution in the Introduction section, we added more detail on how we integrate the two datasets using geospatial information. As you pointed out, this can enhance our contribution because we are able to use comprehensive, consistently defined measures and reliably collected data on both urban agglomeration and innovation, which can help overcome a major obstacle in empirical studies across a large set of countries in the developing world.*

*Since this point is very important, we added the above clarification in the Introduction section, rather than the Methods – Data section. The Data section focuses on the selection of cities which is essential in demonstrating the rigor of the study. And precisely because this detailed discussion of the selection of cities might divert the audience’s attention from the main story and contributions, we put the Data section after the discussion of the results and policy implications, rather than before the results.*

2. The paper could benefit from improvements in readability and presentation. For instance, the introduction of the dataset could be enhanced by providing a more detailed explanation of the variables, particularly the innovation level variable. This would help non-specialist readers better understand the significance of the data and its implications. Additionally, while Table 1 presents descriptive statistics, including mean values, it would be helpful to include explanations or context for these statistics to aid in understanding their significance.

**Response:** *We agree that the original draft did lack a description of the dependent and independent variables before diving into empirical results. We therefore moved some of the descriptions of variables from the Results to the Introduction section, to help readers better gauge the empirical results. Meanwhile, we added more detail on how the dependent and independent variables are constructed. We have edited modestly throughout to improve readability and presentation. For example, when explaining Table 1 in the Results section, we added more context to help readers understand the patterns of innovation and nightlights in our sample.*

In conclusion, "Urban Agglomeration and Innovation in Developing Economies" makes important contributions to the understanding of urban agglomeration and innovation. Enhancing the presentation of the dataset, improving the readability of the paper, and providing more detailed information on the composition of innovation levels would further strengthen its impact and appeal to a wider audience.

*Many thanks for your very constructive comments. They have helped us improve the paper.*

Reviewer #2: This paper attempts to investigate impacts of the costs of excessive agglomeration on innovation in developing economies. Nightlight density is selected to proxy urban economic density and innovation data at the firm level across the developing world is acquired from the WBES. A regression model was proposed to test the relationship between innovation at the firm level and NTL density in the city in which the firm was located, and an interaction term between city population size and NTL density was added to reveal impacts of population on the innovation-NTL relationship. This paper conducted much data analysis, but some crucial questions should be addressed in a major revision.

Major:
1. The title of this paper is “Urban agglomeration and innovation in developing economies”. However, the content is mainly about the relationship between agglomeration costs and urban innovation in developing countries. The scientific question studied and the main findings obtained should be reflected in the title.

**Response:** *In response to this comment, and following your suggestion in point 4 below, we have changed the title to “Agglomeration costs limit sustainable innovation in cities in developing economies”.*

2. In the analysis of experimental results, the failure of urban infrastructure in developing countries to keep pace with population growth has been cited as a reason why large cities in developing countries are not sustainable sources of innovation. The core point is that the conjecture lacks data to support it. It is necessary to collect data related to the infrastructure construction of large cities in developing countries to analyze whether they are really unable to meet the needs of urban residents. At the same time, infrastructure data of cities in developed countries can also be collected to confirm that big cities in developed countries do not have the problem of lack of infrastructure.

**Response:** *Many thanks for the very helpful comment. In order to address the question of infrastructure comparison between developed and developing countries, we collected data related to the infrastructure of large cities (>=5 million population, consistent with the main analysis) in both developing and developed countries. The data are drawn from the Urban Indicators Database published by the UN-Habitat (https://data.unhabitat.org/pages/datasets), which provides information on both urban population and urban transport in cities in developing as well as developed countries. We first identified large cities as those with more than 5 million urban population in 2015 in the UN data, resulting in a sample of 45 cities in developing countries and 14 in developed countries. The Urban Indicators Database provides information on the proportion of urban population that has convenient access to public transport, defined as the estimated share of urban population with access to a public transport stop within a walking distance of 500 meters (for low-capacity public transport systems) or 1000 meters (for high-capacity public transport systems). We note that the UN explicitly links this measure to SDG 11 (https://data.unhabitat.org/pages/urban-transport), which is consistent with our approach. We perform a one-way t-test to compare the mean value of the proportion of urban population that has convenient access to public transport between the group of 45 cities in developing countries and the group of 14 cities in developed countries. We have added a Table 8 in the supporting information to report the results, together with a detailed note beneath the table to explain the details on data sources and measures. Table 8 in the supplementary analysis shows that cities in developing countries have significantly lower proportions of urban population that has convenient access to public transport than cities in developed countries, which supports our conjecture that the large agglomeration costs in cities in developing countries can be associated with the failure of urban infrastructure to keep pace with population growth.*

*We summarize the above key points in the discussion section of the paper. In addition, we point out in the manuscript that our result above is consistent with the existing literature that uses alternative measures of urban infrastructure. For example, Figure 3 (see below) in Zhou et al. (2022) (*[*https://www.pnas.org/doi/10.1073/pnas.2214813119#data-availability*](about:blank#data-availability)*) also shows that infrastructure is much more developed in the Global North than the Global South. We refer to this paper in our manuscript (but do not reproduce the Figure).*

3. Although the use of NTL as an indicator of urban agglomeration costs is theoretically reasonable and innovative, this research method is only applied to developing countries in this paper. Are there studies in developed countries that also use NTL to assess the impact of agglomeration costs on innovation? Are the conclusions about the differences between developed and developing countries influenced by the research method? It is suggested that cities in developed countries should be selected for comparative analysis with the same method.

**Response:** *Thanks for this suggestion. As far as we are aware, there is no research that links agglomeration costs and innovation using NTL in developed countries in a way similar to our paper. However, Mellander et al (2015) apply NTL to study economic activities in Sweden. They conclude that NTL can be useful in developed contexts if applied at a fine-grained geographical level. But they also point out the problem of “saturation” in applying NTL in developed countries. That is, since the maximum value of NTL is restricted to 63, NTL measures can saturate over very bright urban centres: all very bright urban centres may have the same NTL value of 63, even though there are variations in their actual NTL luminosity. Literature has documented that NTL is a better proxy for economic activities in developing countries than developed countries (Chen & Nordhaus, 2011) since top-coding is not a serious problem in most developing cities (Hu & Yao, 2022). We have noted this issue in the “Data” section and explain why top-coding does not impose serious concern for the developing country cities in our paper.*

*At the same time, the WBES database has a very small set of cities in developed countries: 4 in total, Stockholm, Naples, Rome and Tel Aviv (Milan was also surveyed by the WBES but only had 10 firms included in the survey; it was therefore excluded following the selection criteria we use for the main analysis). However, these 4 cities do provide 343 observations of firms. We have performed an analysis on these observations based on the same method we use for cities in developing countries in our main analysis. One slight difference is that instead of using the composite innovation index, we focus on one sub-index–R&D–for cities in developed countries, as it is the closest to the standard measure of innovation, which is patenting, in developed countries. Having new products or new processes–the other two sub-indices in the composite innovation index–might not be innovative to firms in developed countries.*

*Following the model specification for the main analysis, we regress the dummy of whether or not a firm had R&D expenditure on both the linear and square term of logarithm of NTL for the 343 observations in 4 cities in developed countries. Year FE are excluded because there is limited variation in survey years and most of this variation can be captured by region FE. As the dependent variable is a dummy variable rather than an index, we use logit rather than ordered logit models. This model specification is consistent with the one for column 3 in Table 2 in our main analysis. The results for cities in developed cities are shown in column 1 in the following table (Table R1). Unlike the results in column 3 in Table 2, here neither the linear nor the square term of NTL is significant, verifying our conjecture that cities in developed countries and developing countries follow different patterns that link urban agglomeration to innovation. Furthermore, we regress R&D on the linear term of NTL only, without adding the square term. The results in column 2 in Table R1 show that urban agglomeration, proxied by NTL density, is positively and significantly associated with R&D. We further aggregate the results to the city level by plotting the proportion of firms in each of the four cities that had R&D expenditure on the logarithm of NTL density of each city in these developed countries in Figure R1. The dashed line is the linearly fitted line based on regression results. Consistent with column 2 in Table R1, NTL density is positively associated with R&D in these cities.*

*The results in Table R1 and Figure R1 suggest that urban agglomeration monotonically increases innovation in cities in developed countries (as suggested in previous literature), and this is different from the pattern in developing countries where large agglomeration costs associated with excessive agglomeration negatively affect innovation outcomes.*

*In the discussion section of the paper, we provide a brief summary of the key points from the above results. For the moment we put Table R1 and Figure R1 in the response letter only, rather than in the paper or the supplementary analysis, because these results are indicative but not definitive, due to the very small sample and the saturation problem of NTL in cities in developed countries.*

**Table R1 | Innovation and Night Light Intensity for Cities in Developed Countries: Using R&D Input as the Innovation Measure**

|  | (1) | (2) |
| --- | --- | --- |
| VARIABLES | R&D | R&D |
|  |  |  |
| Ln(Night Light) | -1,911.51 | 36.21*** |
|  | (0.15) | (0.00) |
|  | [-4,486.48, 663.46] | [21.29, 51.13] |
| Ln(Night Light) Sqr | 241.07 |  |
|  | (0.14) |  |
|  | [-78.19, 560.33] |  |
| Constant | 3,785.40 | -148.28*** |
|  | (0.15) | (0.00) |
|  | [-1,406.00, 8,976.80] | [-209.00, 87.56] |
|  |  |  |
| Observations | 343 | 343 |
| Method | Logit | Logit |
| Conflict regions | Exclude | Exclude |
| GDPpc>30K countries | Include | Include |
| Region Fixed Effects | Yes | Yes |
| Pseudo R2 | 0.139 | 0.132 |

**Fig. R1 | Innovation and Night Light in Cities in Developed Countries**

References:

Chen, X., Nordhaus, W.D., 2011. Using luminosity data as a proxy for economic statistics. *Proc. of the National Acad. Sci.* 108, 8589–8594

Hsu, F.C., Baugh, K.E., Ghosh, T., Zhizhin, M., Elvidge, C.D., 2015. DMSP-OLS radiance calibrated nighttime lights time series with intercalibration. *Remote Sensing* 7(2), 1855-1876.

Hu, Y., Yao, J., 2022. Illuminating economic growth. *J. Econometrics* 228(2), 359-378.

Mellander, C., Lobo, J., Stolarick, K., Matheson, Z., 2015. Night-time light data: A good proxy measure for economic activity? *PLOS ONE* 10(10), e0139779.

Minor
4. Title. Three principles should be followed for the title: Accurate, Brief, and Clear. A successful title usually states a study's main findings and core conclusions. Please avoid using unnecessary words, such as “A study of/on, Investigation of, Observation on”, etc. I suggest revising your title to “Cost of excessive agglomeration limits sustainable innovation in developing economies”.

**Response:** *As noted above, we have adopted a variant of your suggestion and changed to the title to: “Agglomeration costs limit sustainable innovation in cities in developing economies”. We added the “in cities” as cities are the main focus and units of analysis in this paper.*

5. Abstract: The abstract is a summary of the content of your paper. It is a guide to the most important parts of your study, and it has to be able to stand alone. I recommend writing the abstract following the guideline of the Nature publisher (https://www.nature.com/documents/nature-summary-paragraph.pdf). For your paper, abstract can be a more comprehensive summary of the full text. In addition, please extend the abstract in one paragraph with 200-250 words.

**Response:** *Many thanks for helpful guideline from the Nature publisher. We have now extended the abstract to 238 words following this guideline. We have implemented the following changes:*

*a) We further emphasize the contribution of integrating two datasets to create consistent measures of innovation and urban agglomeration across a large set of developing countries. We made it clear that we rely on nightlight data to measure urban economic density, and we use geospatial information to combine two datasets.*

*b) We added more information on a general context following the Nature guideline. We pointed out that our study suggests that cities in developing countries therefore display different patterns of agglomeration from those documented in the literature focused on developed countries, which echoes the first two sentences in the abstract (which lay out the background and motivation of the study).*

*c) Following the Nature guideline, we added more information on a broader context by linking our study to UN Sustainable Development Goals (SDGs). We made it clear that our analysis highlights the link between SDG 9 which fosters innovation, and SDG 11 which promotes sustainable and resilient cities.*

*d) We re-wrote some sentences to make the explanations clearer. For example, we use “we do not know whether agglomeration affects innovation in the same way” to replace “whether the same is true”.*

6. Keywords. Keywords are a tool to help readers and search engines find your paper. Keywords should contain words and phrases that suggest what the topic is about (the first one or two words). Also include words and phrases that are closely related to your topic (the rest two or three words). Among your keywords, “agglomeration benefits and costs”, is an uncommon phrase, which could be replaced or revised.

**Response:** *Thanks for pointing this out. We changed this keyword to: “urban agglomeration”.*

7. Figure 1. The size of the thematic map and the transparency of the dots should be adjusted to make the information clearer.

**Response:** *We have updated Figure 1 accordingly.*

8. Figure 2. The font on the axis should be larger.

**Response:** *We have updated Figure 2 accordingly.*

*Thank you very much for your most useful and constructive suggestions. They have helped us improve the paper.*

Response to the Journal Office on Journal Requirements

When submitting your revision, we need you to address these additional requirements.

1. Please ensure that your manuscript meets PLOS ONE's style requirements, including those for file naming. The PLOS ONE style templates can be found at

[https://journals.plos.org/plosone/s/file?id=wjVg/PLOSOne_formatting_sample_main_body.pdf](about:blank) and 
[https://journals.plos.org/plosone/s/file?id=ba62/PLOSOne_formatting_sample_title_authors_affiliations.pdf](about:blank)

**Response:** *We have formatted the manuscript based on the two templates provided by the journal office.*

2. For studies involving third-party data, we encourage authors to share any data specific to their analyses that they can legally distribute. PLOS recognizes, however, that authors may be using third-party data they do not have the rights to share. When third-party data cannot be publicly shared, authors must provide all information necessary for interested researchers to apply to gain access to the data. (https://journals.plos.org/plosone/s/data-availability#loc-acceptable-data-access-restrictions)

For any third-party data that the authors cannot legally distribute, they should include the following information in their Data Availability Statement upon submission:
1) A description of the data set and the third-party source
2) If applicable, verification of permission to use the data set
3) Confirmation of whether the authors received any special privileges in accessing the data that other researchers would not have
4) All necessary contact information others would need to apply to gain access to the data

**Response:** *We have included the statements on data availability and code availability in our manuscript to address these requirements.*

3. We note that Figures 1, 4 and Supporting Figure 2 in your submission contain map images which may be copyrighted. All PLOS content is published under the Creative Commons Attribution License (CC BY 4.0), which means that the manuscript, images, and Supporting Information files will be freely available online, and any third party is permitted to access, download, copy, distribute, and use these materials in any way, even commercially, with proper attribution. For these reasons, we cannot publish previously copyrighted maps or satellite images created using proprietary data, such as Google software (Google Maps, Street View, and Earth). For more information, see our copyright guidelines: http://journals.plos.org/plosone/s/licenses-and-copyright.
 
We require you to either (1) present written permission from the copyright holder to publish these figures specifically under the CC BY 4.0 license, or (2) remove the figures from your submission:
 
1) You may seek permission from the original copyright holder of Figures 1, 4 and Supporting Figure 2 to publish the content specifically under the CC BY 4.0 license.  
 
We recommend that you contact the original copyright holder with the Content Permission Form (http://journals.plos.org/plosone/s/file?id=7c09/content-permission-form.pdf) and the following text:
“I request permission for the open-access journal PLOS ONE to publish XXX under the Creative Commons Attribution License (CCAL) CC BY 4.0 (http://creativecommons.org/licenses/by/4.0/). Please be aware that this license allows unrestricted use and distribution, even commercially, by third parties. Please reply and provide explicit written permission to publish XXX under a CC BY license and complete the attached form.”
 
Please upload the completed Content Permission Form or other proof of granted permissions as an ""Other"" file with your submission.
 
In the figure caption of the copyrighted figure, please include the following text: “Reprinted from [ref] under a CC BY license, with permission from [name of publisher], original copyright [original copyright year].”
 
2) If you are unable to obtain permission from the original copyright holder to publish these figures under the CC BY 4.0 license or if the copyright holder’s requirements are incompatible with the CC BY 4.0 license, please either i) remove the figure or ii) supply a replacement figure that complies with the CC BY 4.0 license. Please check copyright information on all replacement figures and update the figure caption with source information. If applicable, please specify in the figure caption text when a figure is similar but not identical to the original image and is therefore for illustrative purposes only.

The following resources for replacing copyrighted map figures may be helpful:

USGS National Map Viewer (public domain): http://viewer.nationalmap.gov/viewer/
The Gateway to Astronaut Photography of Earth (public domain): http://eol.jsc.nasa.gov/sseop/clickmap/
Maps at the CIA (public domain): https://www.cia.gov/library/publications/the-world-factbook/index.html and https://www.cia.gov/library/publications/cia-maps-publications/index.html
NASA Earth Observatory (public domain): http://earthobservatory.nasa.gov/
Landsat: http://landsat.visibleearth.nasa.gov/
USGS EROS (Earth Resources Observatory and Science (EROS) Center) (public domain): http://eros.usgs.gov/#
Natural Earth (public domain): http://www.naturalearthdata.com/

**Response:** *I have written to the journal office on behalf of the authors about this requirement. The journal office confirmed that these figures are compliant with the PLOS policies about copyright already. As requested by the journal office, I copy my original email and the response from the journal office here.*

*Here is my email to the journal office:*

“Dear journal office,

I am writing regarding our submission PONE-D-24-17021, titled "Urban agglomeration and innovation in developing economies". Many thanks for the detailed information on PLOS guidelines. I noticed that one of the requirements is to obtain a CCBY 4.0 license for Figures 1, 4 and supporting Figure 2. As far as I am aware, the sources for these figures are either licensed by CCBY 4.0 already or by open-access data sources that allow for publication at PLOS One. Below are the details.

(1) I drew all the three figures myself using ArcGIS desktop, using the authorized ArcGIS software provided by my university: https://www.lib.sfu.ca/find/other-materials/data-gis/gis/software. I did not use any platform such as Google Map during the process. All the data and code for these three figures are available at: https://github.com/IamAnonymousScholar/Urban-agglomeration-innovation.git. All this information has been provided in the "Data availability" and "Code availability" sections of the original manuscript.

(2) The maps of administrative boundaries used for Figure 1 and 4 are from the open-access source GADM (just the sketch of the map; I drew all the colors and dots myself in ArcGIS). The GADM website explicitly states that their maps can be used for PLOS and no CCBY 4.0 license is required: https://gadm.org/license.html ("Using the data to create maps for publishing of academic research articles is allowed. Thus you can use the maps you made with GADM data for figures in articles published by PLoS, Springer Nature, Elsevier, MDPI, etc".) They mentioned that the map of subnational units in Austria has a different license, but Austria is not in our sample and subnational units in Austria are not included in Figures 1 and 4. The list of cities in our sample is in Table S2 in the supporting information section. The data source GADM as well as its link has been noted in the "Methods - Data" section in the original manuscript.

(3) For Supporting Figure 2, I first downloaded nightlight data from https://doi.org/10.6084/m9.figshare.9828827.v2. I then selected some cities (city maps are from GADM) and combined information on nightlight with information on firms in our sample using ArcGIS. The original nightlight map was in black and white. I changed the color to blue in ArcGIS for better visualization. This nightlight data source is already under CCBY 4.0 (written at the bottom right of the above website). This data source has been cited in the original manuscript and the above link has been provided in the "Data availability" section.

It therefore appears to me that the three figures do not have copyright concerns. If I missed anything, would it be possible to get more detailed information on which exact parts of the above three figures might require further license?”

*Here is the response from the journal office:*

“Dear Dr. Zhang,

According to the sources you've provided, Figs 1 and 4 and the S2 Fig are compliant with PLOS's copyright policy.

When submitting your revised manuscript, please include the clarifications you've provided in your responses to the journal's questions (you can include these responses in the Response to Reviewers file).

I hope this is helpful. Let us know if you have any further questions.

Kind regards,

Teresa

Teresa Diviacchi

Peer Review Operations Specialist

PLOS ONE | plosone@plos.org

Empowering researchers to transform science

1265 Battery Street, Suite 200 San Francisco, CA 94111

Case Number: 08523331

ref:!00DU00Ifis.!500PM0HjNoK:ref”

4. Please include captions for your Supporting Information files at the end of your manuscript, and update any in-text citations to match accordingly. Please see our Supporting Information guidelines for more information: http://journals.plos.org/plosone/s/supporting-information.

**Response:** *We have included the Supporting Information files at the end of the manuscript. All in-text citations are up-to-date.*
